# Promising effects of astaxanthin on pain-related behaviors and morphine tolerance in a mouse model of neuropathic pain: Possible involvement of Nrf2 and the NMDA receptor subunit NR2B

**DOI:** 10.1016/j.neurot.2025.e00765

**Published:** 2025-10-25

**Authors:** Katarzyna Ciapała, Katarzyna Pawlik, Agata Ciechanowska, Wioletta Makuch, Joanna Mika

**Affiliations:** Maj Institute of Pharmacology Polish Academy of Sciences, Department of Pain Pharmacology, 12 Smetna Street, 31-343 Krakow, Poland

**Keywords:** Astaxanthin, Neuropathic pain, Morphine tolerance, Nrf2, NMDA subunit NR2B

## Abstract

The search for new analgesics remains an important issue because therapy for neuropathic pain is still ineffective. Astaxanthin is a naturally derived compound with well-established antiinflammatory and antioxidant properties; however, its pain-relieving mechanisms are not fully understood, and its potential to improve morphine efficacy in neuropathic pain remains poorly defined. This study investigated the effect of astaxanthin on pain-related behaviors in male and female mice, as well as its impact on morphine tolerance and to elucidate the molecular mechanism of its action in neuropathy evoked by chronic constriction injury (CCI) of the sciatic nerve. A single intraperitoneal injection of astaxanthin reduced pain-related behaviors similarly in both sexes after CCI. Importantly, repeated, twice-daily intraperitoneal astaxanthin administration delayed morphine tolerance and prevented motor disturbance. Notably, astaxanthin administered alone induced a long-lasting decrease in hypersensitivity, even to a greater extent than morphine. Western blot results indicated that astaxanthin altered Nrf2 and NR2B proteins expression, suggesting that these factors are involved in analgesic effects of this substance. Pharmacological studies in mice supported these findings: first, the intrathecal administration of trigonelline (an Nrf2 inhibitor) prior to astaxanthin injection caused the loss of its analgesic effect in a CCI model; second, the intrathecal administration of astaxanthin followed by quinolinic acid (an NMDA receptor agonist) prevented the development of hypersensitivity in naive animals. In sum, these results indicate a novel mechanism of analgesic action of astaxanthin, involving Nrf2 and NMDA receptor signaling, and highlight its therapeutic potential in the treatment of neuropathic pain.

## Graphical abstract

**Figure:**
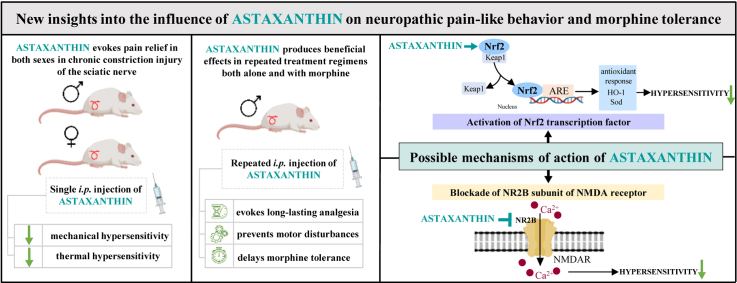

**Intraperitoneal administration of astaxanthin to mice with neuropathy:**

• causes pain relief in males and females after a single injection
• leads to delay of morphine tolerance development
• evokes considerable, longer-lasting pain relief than morphine after repeated treatment
• brings beneficial analgesic properties mediated through Nrf2 and NR2B

## Introduction

Neuropathic pain is a complex and often devastating condition that affects 7–10 ​% of individuals worldwide [1]. It is generally described as a shooting, burning, or electric shock-like sensation. The treatment of neuropathic pain remains challenging, mostly because commonly used medications are only partially effective, and new pharmacological tools have some limitations for use in humans [2]. As a result, management of neuropathic pain typically requires a multimodal approach that combines pharmacological and non-pharmacological strategies. Pharmacological treatment remains the cornerstone of therapy and is based on established guidelines, with first-line agents including certain antidepressants and anticonvulsants [3]. In cases where these methods are insufficient, strong opioids are recommended; however, the rapid development of tolerance necessitates increasing doses, thereby complicating long-term management and increasing the risk of side effects [4]. For these reasons, extended studies to identify new targets as well as effective and safe agents for the treatment of neuropathic pain are constantly needed to provide satisfactory analgesic outcomes and improve patients’ quality of life.

The challenges clinicians encounter in selecting appropriate therapies result primarily from the intricate and still not fully known mechanisms underlying neuropathic pain. Current knowledge indicates the leading roles of not only neurons but also glial cells in this phenomenon, as microglia become rapidly activated after nerve injury and release pro-inflammatory cytokines that enhance neuronal hyperexcitability, while astrocytes contribute to the maintenance and amplification of neuropathic pain [5]. Recent studies have focused on the involvement of different intracellular signaling cascades [[5], [6], [7], [8], [9]]. Studies conducted over the past two decades have highlighted the significant role of mitogen-activated protein kinases (MAPKs) [[10], [11], [12], [13], [14], [15], [16], [17], [18], [19], [20], [21]], which influence sensitization [19,[22], [23], [24], [25]] and opioid effectiveness [[26], [27], [28], [29], [30]]. The MAPK family consists of p38 mitogen-activated protein kinase (p38), extracellular signal-regulated kinase (ERK) and c-Jun N-terminal kinase (JNK) [17,31,32]. To date, several studies have shown that selective MAPK inhibitors attenuate pain-related behaviors in animal models [24,33,34]. In addition, the latest data indicate that among the intracellular mediators of the cellular response, nuclear factor erythroid 2-related factor 2 (Nrf2) or nuclear factor kappa-light-chain-enhancer of activated B cells (NFκB) are other important pharmacological targets for neuropathic pain relief [[35], [36], [37], [38]]. Furthermore, for many years, the N-methyl-d-aspartate (NMDA) receptor has been considered a key player not only in hypersensitivity development [39] but also in the effectiveness of morphine [29,40]. Particular emphasis has been placed on the NR2B subunit of this receptor, since its antagonists may have clinical utility for the treatment of neuropathic pain [41,42]. Oxidative stress has also recently been shown to play a significant role in the development of neuropathic pain, and the use of polymersomes containing superoxide dismutase (Sod) may prevent its onset [43].

As shown above, many different processes and mechanisms may be involved in the development of neuropathic pain, but, interestingly, the compound that seems to influence all the above-mentioned factors is astaxanthin, 3,3′-dihydroxy-beta, beta-carotene-4,4′-dione, which is naturally occurring carotenoid that belong to the xanthophyll family [9]. Astaxanthin may offer potential advantages for treating conditions such as arthritis, diabetes, heart diseases, chronic prostatitis/chronic pelvic pain syndrome, reflux, inflammatory diseases and neurodegenerative illnesses [[44], [45], [46], [47], [48], [49], [50], [51], [52], [53], [54], [55]]. Initial reports from preclinical studies also indicate that astaxanthin may alleviate symptoms of neuropathic pain in rodents; however, these studies were only conducted in males and did not explore the molecular effect of this substance on long-term morphine treatment [[56], [57], [58], [59], [60], [61]]. Previous experiments performed by our team for the first time have shown that a single intrathecal administration of astaxanthin attenuates nerve injury-induced hypersensitivity and enhances the efficacy of morphine [57]. Although several potential mechanisms have been proposed to explain the analgesic effect of astaxanthin, the influences of its repeated administration on the development of hypersensitivity after nerve injury, as well as on the efficacy of morphine in a repeated-dose regimen, remain unclear and these aspects are particularly relevant from a clinical perspective.

The first step of this study was to evaluate and compare the effects of a single intraperitoneal injection of astaxanthin on mechanical and thermal hypersensitivity following chronic constriction injury (CCI) of the sciatic nerve in male and female mice. Subsequently, two pharmacological experiments employing distinct administration protocols were conducted to assess the effects of repeated astaxanthin treatment on pain-related behaviors, the development of morphine tolerance, and motor coordination in CCI-exposed male mice. Furthermore, alterations in the mRNA and/or protein expression levels of cellular markers, such as Iba-1 (microglia/macrophages), Gfap (astrocytes), and cFos (neurons), as well as other factors implicated in neuropathy such as pp38, pERK1/2, pJNK, pNFκB, Nrf2, Sod1 and NR2B, were examined in the spinal cord. The next part of this study focused on the experimental verification of the role of Nrf2 in the analgesic effect of astaxanthin by administering an inhibitor of Nrf2, trigonelline, to male mice subjected to CCI. Moreover, additional behavioral experiments were also performed to evaluate the role of the NR2B subunit of the NMDA receptor in the pain-relieving actions of astaxanthin by administering quinolinic acid, an agonist of this receptor, to naive mice.

## Materials and methods

### Animals

Male and female albino Swiss mice (weighing 20–22 ​g) were purchased from Charles River (Hamburg, Germany). The mice were housed in cages lined with sawdust and maintained on a standard 12-h light/dark cycle (lights on at 6:00 a.m.) with an enriched environment and continuous access to food and water. All the experimental procedures adhered to the guidelines established by the International Association for the Study of Pain and the National Institutes of Health Guide for the Care and Use of Laboratory Animals. Approval was obtained from the II Local Ethics Committee of the Maj Institute of Pharmacology, Polish Academy of Sciences (permit numbers: LKE 75/2023; 18/2023; 153/2023; 242/2024; and 243/2024). In accordance with the 3R principle, all efforts were made to reduce the number of animals used in the experiments and minimize animal suffering.

### Neuropathic pain model

Chronic constriction injury (CCI) of the sciatic nerve was induced in mice under isoflurane anesthesia using the methodology established by Bennett and Xie [62]. An incision was made below the right hip, and the *biceps femoris* and *gluteus superficialis* muscles were carefully separated to expose the sciatic nerve. Three loosely tied ligatures (4/0 silk sutures), spaced 1 ​mm apart, were placed around the nerve distal to the sciatic notch until a brief twitch was observed in the corresponding hind limb. CCI is a well-established procedure that has been performed in our laboratory since 2003 to induce neuropathic pain-related behaviors in rodents [57,[63], [64], [65]]. Compared with control (naive) animals, all mice subjected to CCI exhibited significant mechanical and thermal hypersensitivity, as observed using the von Frey test (p ​< ​0.0001) and the cold plate test (p ​< ​0.0001), at all evaluated time points.

### Behavioral tests

#### von Frey test

Mechanical hypersensitivity was measured using calibrated nylon monofilaments of increasing strength (0.6–6 ​g; with 6 ​g as the cutoff latency) (Ugo Basile, Gemonio, Italy) to observe reactions to mechanical stimuli, as described previously [64]. The mice were habituated in plastic cages with a wire mesh floor 5 ​min before the experiment, and von Frey filaments were applied to the midplantar surface of the hind paw until the limb was lifted. In naive animals, both hind paws were tested in the same manner. This test is used regularly in our laboratory [64,66].

#### Cold plate test

Thermal hypersensitivity was measured using a cold plate analgesia meter (Ugo Basile, Gemonio, Italy), as described previously [64,67]. The temperature of the cold plate was maintained at 2 ​°C, and the cutoff latency was 30 ​s. The mice were positioned on the cold plate separately, and the time to lift the hind paw was noted. In CCI-exposed mice, the injured foot was the first to react to the cold stimulus. In naive mice, both hind paws were observed simultaneously. This test is also used regularly in our laboratory [57,66].

#### Rotarod test

The rotarod test is a frequently used method to assess motor coordination in animals and was used in our previous studies [60]. The mice were placed in separate compartments on a horizontal rod that rotated at an accelerating speed, starting at 2 ​rpm and reaching 40 ​rpm within 300 ​s. The animals were acclimated to the apparatus and trained on the rotating rod. The time until the mouse fell off the rod was recorded. The rotarod test was conducted as a part of the repeated administration study, 1 ​h after morphine administration on Day 11. The cutoff latency was 300 ​s.

### Pharmacological study

#### Substances and routes of administration

The following pharmacological tools were used in our experiments: astaxanthin (A; MedChemExpress, Monmouth Junction, USA), morphine hydrochloride (M; Fagron, Krakow, Poland), trigonelline (T; MedChemExpress), and quinolinic acid (Q; Tocris, Bristol, UK). Substances were administered *via* intrathecal (*i.t.*) or intraperitoneal (*i.p.)* injection. In the case of *i.p.* administration, the control groups for astaxanthin-treated mice received a 30 ​% solution of propylene glycol, and injections were performed in accordance with available guidelines by appropriately adjusting the doses according to the individual body weight measurements [68]. The control groups for morphine-treated mice received water for injection. In case of *i.t* administrations, control groups received the corresponding vehicles: 60 ​% dimethyl sulfoxide for astaxanthin-treated mice and water for injection for trigonelline or quinolinic acid-treated groups. This route of administration is a standard procedure in our laboratory [57,69]. The procedure was performed using a Hamilton syringe fitted with a fine-gauge needle, in accordance with established protocols described previously [70]. The injections were delivered in the lumbar segment of the spinal cord (between the L5 and L6 vertebrae) in a volume of 5 ​μl, with the tail reflex serving as an indicator of successful administration. All control groups were tested at the same time points as the corresponding experimental groups.

#### Dose dependency study

Astaxanthin was administered *i.p.* at doses of 1, 10, 25 or 50 ​mg/kg on Day 7 after CCI to both male and female mice. These doses were selected based on our previous studies and the available literature [57,60,71]. The behavioral tests were conducted 0.5, 1.5, 3, 5 and 24 ​h after these injections.

#### Repeated astaxanthin administration in CCI-exposed mice after the development of morphine tolerance

Experiments were conducted in male mice subjected to CCI on Day 0 to evaluate the effects of astaxanthin on pain-related behaviors in animals that had developed morphine tolerance. Beginning on Day 1, mice received *i.p*. injections of either vehicle or morphine (30 ​mg/kg) twice daily, in the morning and afternoon (schemes A on Fig. 2, Fig. 3). Behavioral assessments were performed every second day, 30 ​min after the morning injection. On Day 8, a decrease in morphine effectiveness was noted. On the morning of Day 9, morphine-treated animals were divided into two groups: one received the vehicle and the other received astaxanthin (25 ​mg/kg, *i.p*.), followed by morphine administration 30 ​min later in both groups. Similarly, animals that had received vehicle treatment up to Day 8 were divided into two groups on Day 9: one received astaxanthin followed by vehicle 30 ​min later, while the other group received vehicle at both time points, with the same interval between administrations. Behavioral testing was conducted 30 ​min after the second injection. The afternoon injection schedule remained the same, although no behavioral testing was performed. This regimen of drug administration and testing continued for the next 8 days.

#### Repeated administration of astaxanthin with morphine in CCI-exposed mice

Experiments were conducted in male mice subjected to CCI, using an alternative administration regimen to evaluate whether astaxanthin administered prior to the onset of morphine tolerance delays or prevents the development of this phenomenon (schemes A on Fig. 4, Fig. 5). Tolerance development was evaluated by administering either vehicle or morphine (30 ​mg/kg, *i.p.*) twice daily for 14 days, starting on the first day after CCI, following a previously described protocol [72,73]. In the experimental groups, vehicle or astaxanthin (25 ​mg/kg, *i.p.*) was administered 16 and 1 ​h prior to CCI, and subsequently twice daily, 30 ​min before each vehicle or morphine injection, for the following 14 days. Behavioral testing was conducted daily, 30 ​min after the morning administration of vehicle or morphine. Additionally, on Day 11, the rotarod test was performed to assess motor coordination of the animals.

**Fig. 1:**
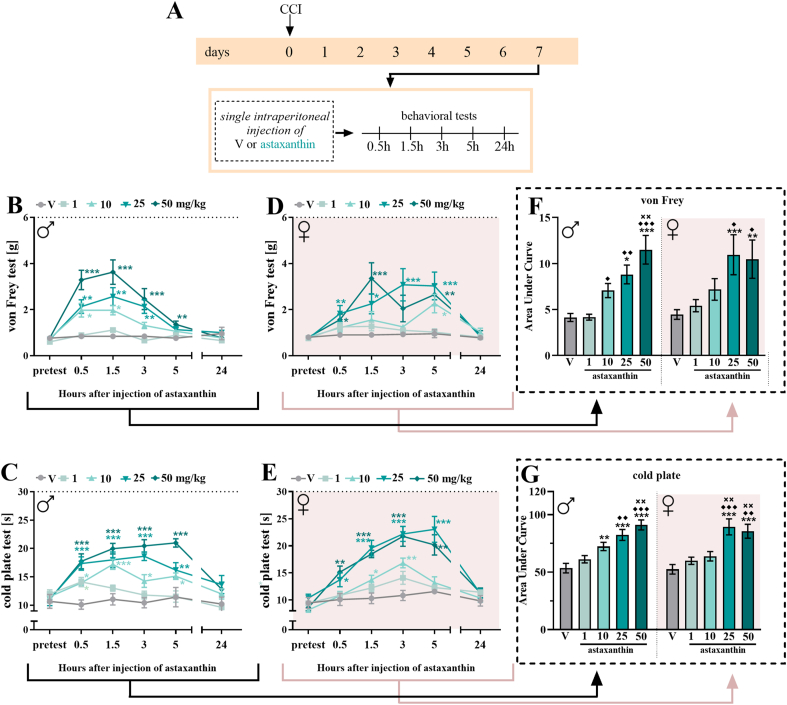
**Influence of*****i.p.*****astaxanthin administration on pain-related behaviors in male and female mice exposed to CCI. The effects of injections of vehicle and astaxanthin (1, 10, 25 and 50 mg/kg) on mechanical and thermal hypersensitivity were assessed at 0.5, 1.5, 3, 5 and 24 h after treatment on the 7th day after CCI (A) in males (B, C) and females (D, E).** The data are presented as the means ± SEMs, and the numbers of animals are as follows: males, V = 5, 1 mg/kg = 7, 10 mg/kg = 7, 25 mg/kg = 6, and 50 mg/kg = 7; females, V = 8, 1 mg/kg = 8, 10 mg/kg = 8, 25 mg/kg = 8, and 50 mg/kg = 8. Additionally, the results obtained from the von Frey **(B, D)** and cold plate (**C, E)** tests were analyzed by calculating the areas under the curves **(F, G)** to visualize overall changes in the efficacy of the different doses. The intergroup differences were analyzed using one-way ANOVA with Bonferroni's multiple comparisons *post hoc* test. ∗p < 0.05, ∗∗p < 0.01 and ∗∗∗p < 0.001 indicate differences between V-treated CCI mice and substance-treated CCI mice; ♦p < 0.05, ♦♦p < 0.01 and ♦♦♦p < 0.001 indicate differences between animals treated with astaxanthin at a dose of 1 mg/kg; p < 0.01 indicates differences between animals treated with astaxanthin at a dose of 10 mg/kg. The dotted lines indicate the cutoff values for the tests. Abbreviations: V, vehicle; CCI, chronic constriction injury.

**Fig. 2:**
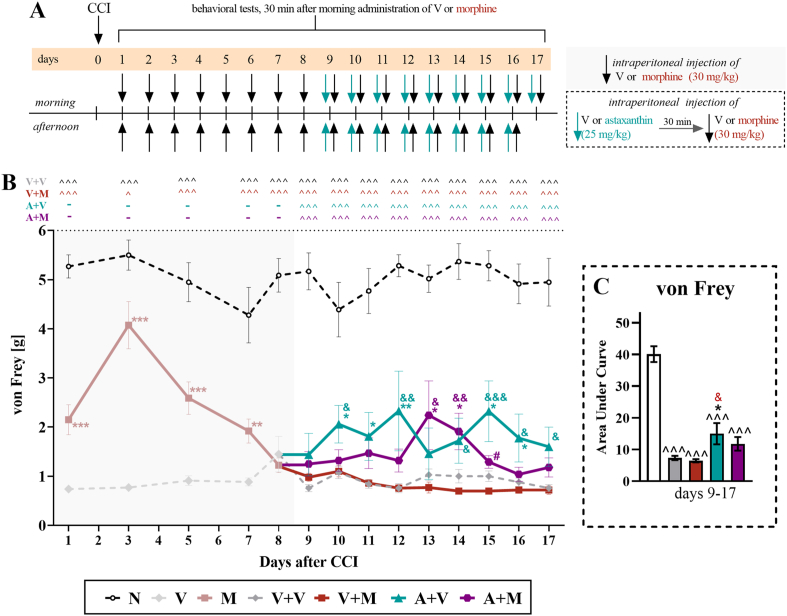
**The influence of the repeated twice daily administration of vehicleand astaxanthin (25 mg/kg,*****i.p.*****) that started when the repeated twice daily treatment with vehicle or morphine (30 mg/kg, i.p.) lost its efficacy on mechanical hypersensitivity in male mice with CCI**. On the 1st day after CCI, vehicle or morphine was administered twice daily until Day 8, then the experimental groups were divided, and for the next 9 days, until Day 17, the mice received vehicle or astaxanthin twice daily 30 min before treatment with the vehicle or morphine (**A**). The von Frey tests (**B**) were conducted 30 min after the vehicle or morphine injection. The data are presented as the means ± SEMs, and the numbers of animals analyzed until Day 8 were as follows: N = 10; V = 20; and M = 20. The numbers of animals analyzed from Days 9–17 were as follows: N = 10; V + V = 10; V + M = 10; A + V = 9–10; and A + M = 10. The results were analyzed using one-way ANOVA with Bonferroni's multiple comparisons *post hoc* test. Additionally, the data obtained from the von Frey **(B)** test were analyzed as area under the curve **(C)** to visualize overall changes between treatments. ˆp < 0.05 and ˆˆˆp < 0.001 indicate differences between naive and CCI-exposed mice; ∗p < 0.05, ∗∗p < 0.01 and ∗∗∗p < 0.001 indicate differences compared with V + V-treated CCI-exposed mice; &p < 0.05, &&p < 0.01 and &&&p < 0.001 indicate differences compared with the V + M-treated CCI-exposed mice; and #p < 0.05 indicates differences compared with the A + V-treated CCI-exposed mice. The dotted lines indicate the cutoff values for the test. Abbreviations: A, astaxanthin; M, morphine; N, naive; V, vehicle; CCI, chronic constriction injury.

**Fig. 3:**
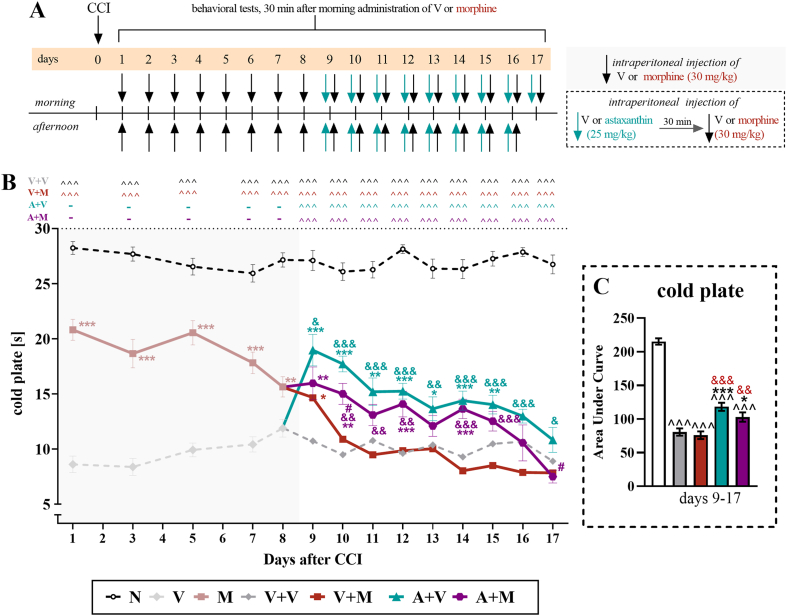
**The influence of the repeated twice daily administration of vehicle and astaxanthin (25 mg/kg,*****i.p.*****) that started whenrepeated twice daily treatment with vehicle or morphine (30 mg/kg,*****i.p*****.) lost its efficacy on thermal hypersensitivity in male mice with CCI.** On the 1st day after CCI, vehicle or morphine was administered twice daily until Day 8, then the experimental groups were divided, and for the next 9 days, until Day 17, the mice received vehicle or astaxanthin twice daily 30 min before treatment with the vehicle or morphine (**A**). The cold plate tests (**B**) were conducted 30 min after the vehicle or morphine injections. The data are presented as the means ± SEMs, and the numbers of animals analyzed until Day 8 were as follows: N = 10; V = 20; and M = 20. The numbers of animals analyzed from Days 9–17 were as follows: N = 10; V + V = 10; V + M = 10; A + V = 9–10; and A + M = 10. The results were analyzed using one-way ANOVA with Bonferroni's multiple comparisons *post hoc* test. Additionally, the data obtained from the cold plate **(B)** test were analyzed as area under the curve **(C)** to visualize overall changes between treatments. ^^^p<0.001 indicates differences between naive and CCI-exposed mice; ∗p < 0.05, ∗∗p < 0.01 and ∗∗∗p < 0.001 indicate differences compared with V + V-treated CCI-exposed mice; &p < 0.05, &&p < 0.01, and &&&p < 0.001 indicate differences compared with the V + M-treated CCI-exposed mice; and #p < 0.05 indicates differences compared with the A + V-treated CCI-exposed mice. The dotted lines indicate the cutoff values for the test. Abbreviations: A, astaxanthin; M, morphine; N, naive; V, vehicle; CCI, chronic constriction injury.

**Fig. 4:**
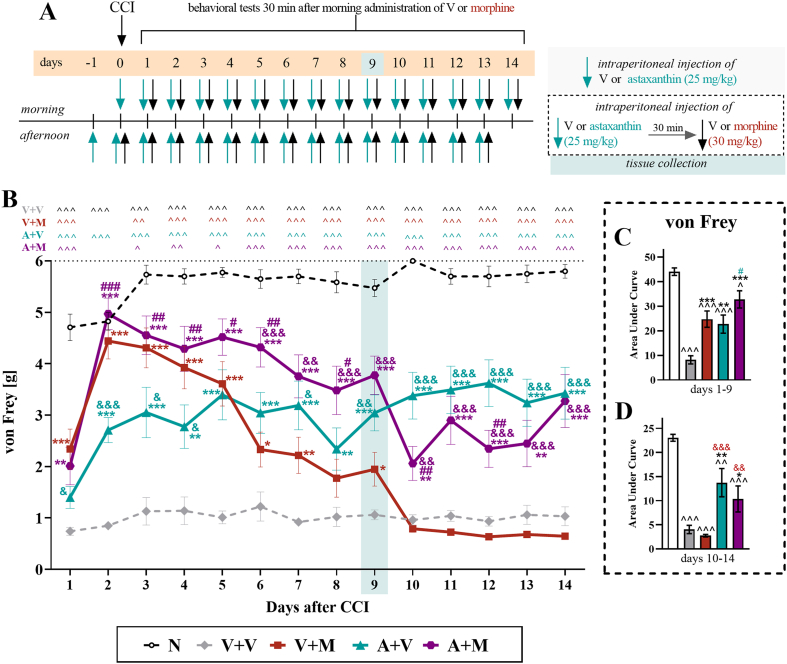
**The influence of repeated twice daily intraperitoneal injections of the vehicle, morphine (30 mg/kg*****i.p*****.), astaxanthin(25 mg/kg*****i.p.*****) or the combination of astaxanthin and morphine on mechanical hypersensitivity in male mice with CCI.** The vehicle and astaxanthin were repeatedly administered 16 and 1 h before CCI and then twice daily for 14 days 30 min before treatment with the vehicle or morphine **(A)**. The von Frey tests (**B**) were conducted 30 min after the vehicle or morphine injection. The data are presented as the means ± SEMs, and the numbers of animals were as follows: N = 10–20; V + V = 19–20; V + M = 18–20; A + V = 19–20; and A + M = 19–20. The results were analyzed using one-way ANOVA with Bonferroni's multiple comparisons *post hoc* test. Additionally, the data obtained from the von Frey **(B)** test were analyzed as areas under the curves **(C, D)** to visualize overall changes between treatments at two time intervals. ^p <0.05, ^^p <0.01, and ^^^p<0.001 indicate differences between naive and CCI mice; ∗p < 0.05, ∗∗p < 0.01 and ∗∗∗p < 0.001 indicate differences compared with V + V-treated CCI mice; &p < 0.05, &&p < 0.01, and &&&p < 0.001 indicate differences compared with the V + M-treated CCI mice; and #p < 0.05, ##p < 0.01, and ###p < 0.001 indicate differences compared with the A + V-treated CCI mice. The dotted lines indicate the cutoff values for the tests, and the blue rectangles represent the time points of tissue collection. Abbreviations: A, astaxanthin; M, morphine; N, naive; V, vehicle; CCI, chronic constriction injury.

**Fig. 5:**
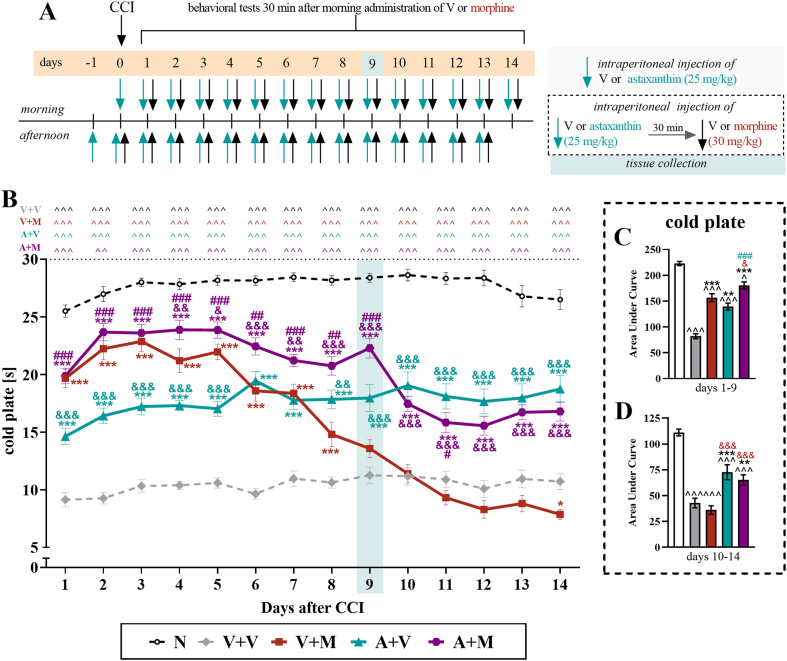
**The influence of repeated twice daily intraperitoneal injections of the vehicle, morphine (30 mg/kg*****i.p*****.), astaxanthin (25 mg/kg*****i.p.*****) or the combination of astaxanthin and morphine on thermal hypersensitivity in male mice with CCI**. The vehicle and astaxanthin were repeatedly administered 16 and 1 h before CCI and then twice daily for 14 days 30 min before treatment with the vehicle or morphine **(A)**. The cold plate tests (**B**) were conducted 30 min after vehicle or morphine injection. The data are presented as the means ± SEMs, and the numbers of animals were as follows: N = 10–20; V + V = 19–20; V + M = 18–20; A + V = 19–20; and A + M = 19–20. The results were analyzed using one-way ANOVA with Bonferroni's multiple comparisons *post hoc* test. Additionally, the data obtained from the cold plate **(B)** test were analyzed as areas under the curves **(C, D)** to visualize overall changes between treatments at two time intervals. ^p<0.05, ^^p<0.01 and ^^^p<0.001 indicate differences between naive and CCI-exposed mice; ∗p < 0.05, ∗∗p < 0.01 and ∗∗∗p < 0.001 indicate differences compared with V + V-treated CCI-exposed mice; &p < 0.05, &&p < 0.01, and &&&p < 0.001 indicate differences compared with the V + M-treated CCI-exposed mice; and #p < 0.05, ##p < 0.01, and ###p < 0.001 indicate differences compared with the A + V-treated CCI-exposed mice. The dotted lines indicate the cutoff values for the test, and the blue rectangles represent the time points of tissue collection. Abbreviations: A, astaxanthin; M, morphine; N, naive; V, vehicle; CCI, chronic constriction injury.

#### A single intrathecal injection of astaxanthin preceded by a trigonelline injection in CCI-exposed mice

To determine whether the analgesic effect of astaxanthin in neuropathic pain depends on the transcription factor Nrf2, male mice were administered its inhibitor, trigonelline (5 μg/5 ​μl, *i.t*.), or vehicle on Day 7 after CCI. Then, 30 ​min later, vehicle or astaxanthin (2 μg/5 ​μl, *i.t*.) was administered, and behavioral assessments were conducted at 0.5, 1.5, 3, 5, and 24 ​h after the last injection (scheme A on Fig. 10). The dose of trigonelline was selected based on a previously published study [74].

#### A single intrathecal quinolinic acid injection preceded by an astaxanthin injection in naive mice

To assess whether the analgesic effects of astaxanthin are mediated through mechanisms involving the NMDA receptor, naive mice received a single *i.t*. injection of the NMDA receptor agonist quinolinic acid (10 μg/5 ​μl) or vehicle. This injection was preceded by a single *i.t.* administration of vehicle or astaxanthin (2 μg/5 ​μl), given 30 ​min earlier. Behavioral assessments were performed at 0.5, 1.5, 3, 5, and 24 ​h after the administration of quinolinic acid (scheme A on Fig. 11). The dose of quinolinic acid was selected based on our preliminary experiments.

### Analysis of gene expression *via* reverse transcription quantitative real-time polymerase chain reaction

Immediately following decapitation, the ipsilateral (right) lumbar spinal cord segments (L4–L6) were dissected from CCI-exposed mice on Day 9 after CCI, approximately 4 ​h after the administration of vehicle or morphine. In the case of naive animals, the corresponding spinal cord regions were collected at the same time point to ensure consistency across experimental conditions. In accordance with the Chomczynski and Sacchi protocol [75], total RNA was extracted using TRIzol reagent (Invitrogen, Carlsbad, USA). The quality and concentration of RNA were checked using a DeNovix DS-11 spectrophotometer (DeNovix Inc., Wilmington, USA). RNase inhibitor (Promega, Mannheim, Germany), an Omniscript RT Kit (Qiagen Inc., Hilden, Germany) and oligo (dT16) primers (Qiagen Inc.) were used to perform reverse transcription of 1 ​μg of total RNA at 37 ​°C. Next, the obtained cDNA templates were diluted 1:10 with RNase-/DNase-free H_2_O. RT‒qPCR was performed with approximately 50 ​ng of cDNA template from each sample using Assay-On-Demand TaqMan probes (Applied Biosystems, Foster City, USA) and an iCycler device (Bio-Rad, Hercules, Warsaw, Poland). The following TaqMan primers were used: *Iba-1* (Mm00479862_g1), *Gfap* (Mm01253033_m1), *cFos* (Mm00487425_m1), *Sod1* (Mm01302428_m1), *Nrf2* (Mm00477784_m1), *Grin2b* (Mm00433820_m1) and *Hprt* (Mm00446968_m1). *Hprt* was used as an endogenous control and an adequate housekeeping gene. The cycle threshold values were automatically calculated using CFX Manager v.2.1 software (Bio-Rad) with the default parameters. The RNA content was calculated using formula 2^−(threshold cycle)^.

### Analysis of protein levels *via* Western blotting

Immediately following decapitation, the ipsilateral (right) lumbar spinal cord segments (L4–L6) were dissected from CCI-exposed mice on Day 9, approximately 6 ​h after last substances administration. In the case of naive animals, the corresponding spinal cord regions were collected at the same time point to ensure consistency across experimental conditions. The samples were then placed in RIPA buffer supplemented with a protease/phosphatase inhibitor cocktail (Sigma‒Aldrich, St. Louis, USA), homogenized and cleared *via* centrifugation (14 ​000 ​rpm, 30 ​min at 4 ​°C). The total protein concentration was determined using the bicinchoninic acid method. The obtained samples (10 ​μg of protein) were heated in a mixture of loading buffer (4x Laemmli Buffer, Bio-Rad) and 2-mercaptoethanol (Bio-Rad) for 8 ​min at 98 ​°C. Using 4–15 ​% Criterion™ TGX™ precast polyacrylamide gels (Bio-Rad), electrophoresis was performed. Next, the proteins were transferred (semidry transfer at 25 ​V for 30 ​min) to Immune-Blot PVDF membranes (Bio-Rad) and blocked at RT for 1 ​h with 5 ​% bovine serum albumin (Sigma‒Aldrich) in Tris-buffered saline containing 0.1 ​% Tween-20 (TBST). Following the blocking step, the membranes were washed with TBST and incubated overnight (4 ​°C) with the following primary antibodies: rabbit anti-cFos (1:1000, 2250; Cell Signaling, Danvers, USA), anti-Iba-1 (1:500, NBP2-19019; Novus, Abingdon, UK), anti-Gfap (1:10 ​000, NB300-141; Novus), anti-pp38 (1:1000, 4631; Cell Signaling), anti-pERK1/2 (1:1000, 4370; Cell Signaling), anti-pJNK (1:1000, 4671; Cell Signaling), anti-pNFκB (1:1000, sc-33039; Santa Cruz, Dallas, USA), anti-Sod1 (1:1000, 37 ​385; Cell Signaling), anti-Nrf2 (1:1000, 20 ​733; Cell Signaling), anti-NR2B (1:1000, 4212; Cell Signaling) and mouse anti-β-actin (1:1000; SAB1305554-40TST, Sigma). Afterward, the membranes were washed with TBST and incubated for 1 ​h at room temperature with HRP-conjugated anti-rabbit or anti-mouse secondary antibodies (1:5000, Vector Laboratories, Burlingame, CA, USA) diluted in SignalBoost™ Immunoreaction Enhancer Kit (Merck) buffer. Proteins were detected using Clarity™ Western ECL Substrate (Bio-Rad) and visualized with a Fujifilm LAS-4000 FluorImager system. Fujifilm MULTI GAUGE software V3.0 (Tokyo, Japan) was used to estimate the intensities of the immunoreactive bands.

### Data analysis

The behavioral data are presented as the means ​± ​SEMs in grams or seconds. One-way analysis of variance (ANOVA) was used to evaluate the experimental results, followed by Bonferroni *post hoc* correction of selected pairs measured separately at each time point. The results shown in Fig. 1, Fig. 2, Fig. 3, Fig. 4, Fig. 5, Fig. 10, Fig. 11 were additionally evaluated using two-way repeated-measures ANOVA to detect time‒drug interactions. Moreover, the area under the curve (AUC) was calculated for the behavioral data by trapezoidal and Simpson's rules, as described by Tallarida and Murray [76], and a *t*-test was used to compare the effects of the tested doses between the sexes (Fig. 1). For the dose dependency study, the Litchfield and Wilcoxon methods were applied to determine the antinociceptive dose necessary to produce a 50 ​% response (ED_50_) and the 95 ​% confidence intervals of the quantitative data, which were automatically calculated with Pharm/PCS software (version 4) for the results obtained 1.5 ​h after drug administration. For biochemical analyses, the RT‒qPCR and Western blotting results are presented as the fold changes relative to the control (naive) ​± ​SEMs. The number of animals was chosen based on our previous research and is noted in the figure captions [66,77,78]. Similar to the behavioral studies, the data were analyzed using one-way analysis of variance followed by Bonferroni *post hoc* correction. All the statistical analyses and graphs were generated using Prism software (version 9.1.2 (226), GraphPad Software, Inc., San Diego, USA). Differences were considered significant when p ​< ​0.05.

## Results

### Effects of a single intraperitoneal administration of astaxanthin on mechanical and thermal hypersensitivity in male and female mice on the 7th day after CCI

Single *i.p.* injections of astaxanthin at different doses (1, 10, 25 and 50 ​mg/kg) were performed in male and female mice on Day 7 after CCI (Fig. 1A), and the influence of the substance on hypersensitivity to mechanical (Fig. 1B–D) and thermal (Fig. 1C–E) stimuli was measured.

According to the von Frey test, astaxanthin reduced mechanical hypersensitivity in both sexes. In male mice, the most effective dose of this substance was 50 ​mg/kg, which had an analgesic effect on mechanical hypersensitivity at 0.5 ​h (F_4,27_ ​= ​12.74; p ​< ​0.0001), 1.5 ​h (F_4,27_ ​= ​11.95; p ​< ​0.0001), 3 ​h (F_4,27_ ​= ​8.86; p ​= ​0.0001) and 5 ​h (F_4,27_ ​= ​3.34; p ​= ​0.0240) after treatment (Fig. 1B). A dose of 25 ​mg/kg had an analgesic effect at the same time points, except 5 ​h, whereas the other dose of 10 ​mg/kg caused a decrease in mechanical hypersensitivity only at 0.5 ​h and 1.5 ​h after treatment. The analgesic effects of these doses were no longer observed 24 ​h after administration, and the lowest dose of 1 ​mg/kg had no effect at any of the tested time points (Fig. 1B). Two-way repeated-measures ANOVA confirmed a significant interaction between the investigated treatment and the tested time points (F_20,162_ ​= ​5.35; p ​< ​0.0001, Fig. 1B). In female mice, the highest dose of astaxanthin (50 ​mg/kg) also reduced mechanical hypersensitivity, with the most pronounced effect observed 1.5 ​h (F_4,35_ ​= ​5.57; p ​= ​0.0014) after administration (Fig. 1D). The dose of 25 ​mg/kg reduced mechanical hypersensitivity at all assessed time points, except 24 ​h, with the greatest effect seen at 3 ​h (F_4,35_ ​= ​4.63; p ​= ​0.0134) and 5 ​h (F_4,35_ ​= ​5.77; p ​= ​0.0134) after administration. The other doses did not cause a decrease in mechanical hypersensitivity at any time point after treatment, except for the dose of 10 ​mg/kg at 5 ​h after administration. Two-way repeated-measures ANOVA confirmed a significant interaction between the investigated treatment and the tested time points (F_20,210_ ​= ​2.78; p ​= ​0.0001, Fig. 1D). Moreover, the analysis of the AUC revealed that the same dose of *i.p.* administered astaxanthin had a similar effect on hypersensitivity to mechanical stimuli in both male and female mice subjected to CCI (Fig. 1F).

According to the cold plate test results, astaxanthin also reduced thermal hypersensitivity in both sexes. In males, the best analgesic effects were observed for a dose of 50 ​mg/kg at 5 ​h (F_4,27_ ​= ​14.49; p ​< ​0.0001); however, this dose was also effective at other time points tested, except 24 ​h after administration (Fig. 1C). Similarly, the dose of 25 ​mg/kg also effectively reduced thermal hypersensitivity, with the most pronounced effect observed 3 ​h (F_4,27_ ​= ​19.99; p ​< ​0.0001) after administration. The 10 ​mg/kg dose had a slightly weaker effect; however, it also diminished thermal hypersensitivity at each time point except 24 ​h, whereas the 1 ​mg/kg dose reduced thermal hypersensitivity only at 0.5 ​h (F_4,27_ ​= ​8.54; p ​= ​0.0001) after treatment. Two-way repeated-measures ANOVA confirmed a significant interaction between the investigated treatment and the tested time points (F_20,162_ ​= ​3.53; p ​< ​0.0001, Fig. 1C). In female mice, the highest doses of astaxanthin (25 and 50 ​mg/kg) were similarly effective and significantly reduced thermal hypersensitivity at all assessed time points, except at 24 ​h post-administration. The most pronounced effects were observed between 1.5 ​h (F_4,35_ ​= ​16.85; p ​< ​0.0001) and 5 ​h (F_4,35_ ​= ​12.77; p ​< ​0.0001) after administration (Fig. 1E). The 10 ​mg/kg dose produced the strongest analgesic effect at 3 ​h (F_4,35_ ​= ​14.26; p ​< ​0.0001) post-administration, while the 1 ​mg/kg dose did not elicit any significant effect. Two-way repeated-measures ANOVA confirmed a significant interaction between the investigated treatment and the tested time points (F_20,210_ ​= ​4.62; p ​< ​0.0001, Fig. 1E). Moreover, an analysis of the AUC revealed that after *i.p.* administration, astaxanthin had a similar effect on hypersensitivity to thermal stimuli in both male and female mice with neuropathic pain (Fig. 1G).

As specified by the calculated ED_50_ for *i.p.* administration, the values obtained from the von Frey test were the same for both sexes. In the cold plate test, a lower ED_50_ value was calculated (Table 1), but these differences were not statistically significant compared with those for females. Due to the slightly better effect of astaxanthin administered at a dose of 50 ​mg/kg on males, experiments with repeated administration of this substance were performed to obtain a daily dose of 50 ​mg/kg, which was achieved by two injections of 25 ​mg/kg astaxanthin.

**Table 1:** The ED_50_ values for the effects of *i.p.* (1, 10, or 50 mg/kg) astaxanthin administered to male and female mice were calculated, along with 95 % confidence intervals for CCI-exposed mice, as measured by von Frey and cold plate tests 1.5 h after the drug injection.

ASTAXANTHIN		
BEHAVIORAL TESTS	ED_50_	
	♂	♀
von Frey	130.62 (4.13–4129.05)	130.62 (4.13–4129.05)
cold plate	3.34 (0.36–30.68)	12.50 (1.18–132.58)

### Effects of repeated intraperitoneal injections of astaxanthin on the mechanical and thermal hypersensitivity of CCI-exposed male mice that developed morphine tolerance

An investigation of the effect of astaxanthin on mechanical hypersensitivity in animals that had developed tolerance to morphine was performed in male mice subjected to CCI (Fig. 2A), in accordance with the regimen provided in Materials and methods. The results indicated that vehicle-treated mice subjected to CCI exhibited persistent mechanical hypersensitivity throughout the 17-day experimental period. In the von Frey test, 30 ​mg/kg morphine administered from Day 1 after CCI lost its effectiveness in reducing hypersensitivity to mechanical stimuli on Day 8 (F_2,47_ ​= ​38.84; p ​< ​0.0001) (Fig. 2B). The administration of astaxanthin at the time of the loss of the analgesic effect of morphine improved the effect of morphine on Days 13 (F_4,45_ ​= ​17.11; p ​< ​0.0001) and 14 (F_4,44_ ​= ​37.15; p ​< ​0.0001) after CCI. In the animals that received astaxanthin alone, a slight analgesic effect was observed until the last day of the experiment. Additionally, the analysis of the AUC of the von Frey test (Fig. 2C) revealed that only astaxanthin administered alone had a beneficial effect on mechanical hypersensitivity in CCI animals (F_4,20_ ​= ​42.40; p ​< ​0.0001).

An investigation of the effect of astaxanthin on thermal hypersensitivity in animals that had developed tolerance to morphine was performed in male mice subjected to CCI (Fig. 3A), in accordance with the regimen described in Materials and methods. The results indicated that vehicle-treated mice subjected to CCI exhibited persistent thermal hypersensitivity throughout the 17-day experimental period. In the cold plate test, 30 ​mg/kg morphine administered from Day 1 after CCI lost its effectiveness in reducing hypersensitivity to thermal stimuli on Day 10 (F_4,45_ ​= ​52.05; p ​< ​0.0001) (Fig. 3B). Compared with morphine alone, the administration of astaxanthin at the time of weaker morphine analgesic action significantly improved the reduction in thermal hypersensitivity until Day 17 (F_4,44_ ​= ​110.30; p ​< ​0.0001); however, during the last 2 days of the experiments, both groups exhibited similar responses in the cold plate test. Moreover, the animals that received astaxanthin alone presented the greatest reduction in thermal hypersensitivity, and this effect persisted throughout the experiment. Additionally, the analysis of the AUC of the cold plate test (Fig. 3C) indicated that astaxanthin administered alone and astaxanthin administered with morphine had beneficial effects on thermal hypersensitivity in CCI-exposed animals (F_4,20_ ​= ​97.41; p ​< ​0.0001).

### Effects of repeated intraperitoneal injections of astaxanthin with morphine on the mechanical and thermal hypersensitivity of CCI-exposed male mice

An investigation of the influence of astaxanthin on the development of morphine tolerance to mechanical hypersensitivity was performed in male mice subjected to CCI (Fig. 4A), in accordance with the regimen provided in Materials and methods. The results of the von Frey test indicated that vehicle-treated CCI mice exhibited mechanical hypersensitivity until the last, 14th day of the experiment. Morphine was administered at a dose of 30 ​mg/kg starting on Day 0, the day of CCI, and a progressive decline in its analgesic efficacy was observed, with no effect detected on Day 10 (F_4,83_ ​= ​39.22; p ​< ​0.0001) (Fig. 4B). Pretreatment with astaxanthin followed by the 14-day administration of this substance (twice daily, 25 ​mg/kg *i.p.*) with morphine (twice daily, 30 ​mg/kg, *i*.*p.*) significantly improved the analgesic effects of this opioid, as observed in the von Frey test starting from the 6th day after CCI (F_4,93_ ​= ​27.61; p ​< ​0.0001). Additionally, the injection of astaxanthin alone attenuated the development of mechanical hypersensitivity in mice from the second day following CCI until the last day of the experiment. Moreover, the analysis of the AUC of the von Frey test (Fig. 4C and D), which was calculated for two selected intervals (Days 1–9 and 10–14), indicated that morphine significantly lost its effectiveness over time, whereas animals receiving astaxanthin alone or astaxanthin with morphine continued to display significantly reduced hypersensitivity to mechanical stimuli compared with the vehicle-treated mice (F_4,20_ ​= ​19.69; p ​< ​0.0001).

An investigation of the influence of astaxanthin on the development of morphine tolerance to thermal hypersensitivity was performed in male mice subjected to CCI (Fig. 5A), in accordance with the regimen provided in Materials and methods. The results of the cold plate test indicated that vehicle-treated CCI mice exhibited thermal hypersensitivity until the last, 14th day of the experiment. Morphine was administered at a dose of 30 ​mg/kg starting on Day 0, the day of CCI, and a progressive decline in its analgesic efficacy was observed, with no effect on thermal hypersensitivity detected on Day 9 (F_4,94_ ​= ​69.60; p ​< ​0.0001) (Fig. 5B). Pretreatment with astaxanthin followed by the 14-day administration of this substance (twice daily, 25 ​mg/kg *i.p.*) with morphine (twice daily, 30 ​mg/kg, *i*.*p.*) significantly improved the analgesic effects of this opioid, as observed in the cold plate test until Day 14 (F_4,80_ ​= ​58.44; p ​< ​0.0001). Additionally, the injection of astaxanthin alone attenuated the development of thermal hypersensitivity in mice following CCI until the last day of the experiment. Moreover, the analysis of the AUC of the cold plate test (Fig. 5C and D), which was calculated for two selected intervals (Days 1–9 and 10–14), indicated that morphine significantly lost its effectiveness over time, whereas animals receiving astaxanthin alone or astaxanthin with morphine continued to have significantly reduced hypersensitivity to thermal stimuli compared with the vehicle-treated mice (F_4,20_ ​= ​34.95; p ​< ​0.0001).

### Influence of repeated intraperitoneal injections of astaxanthin and morphine on the motor coordination and body weight of male mice with CCI

**Fig. 6:**
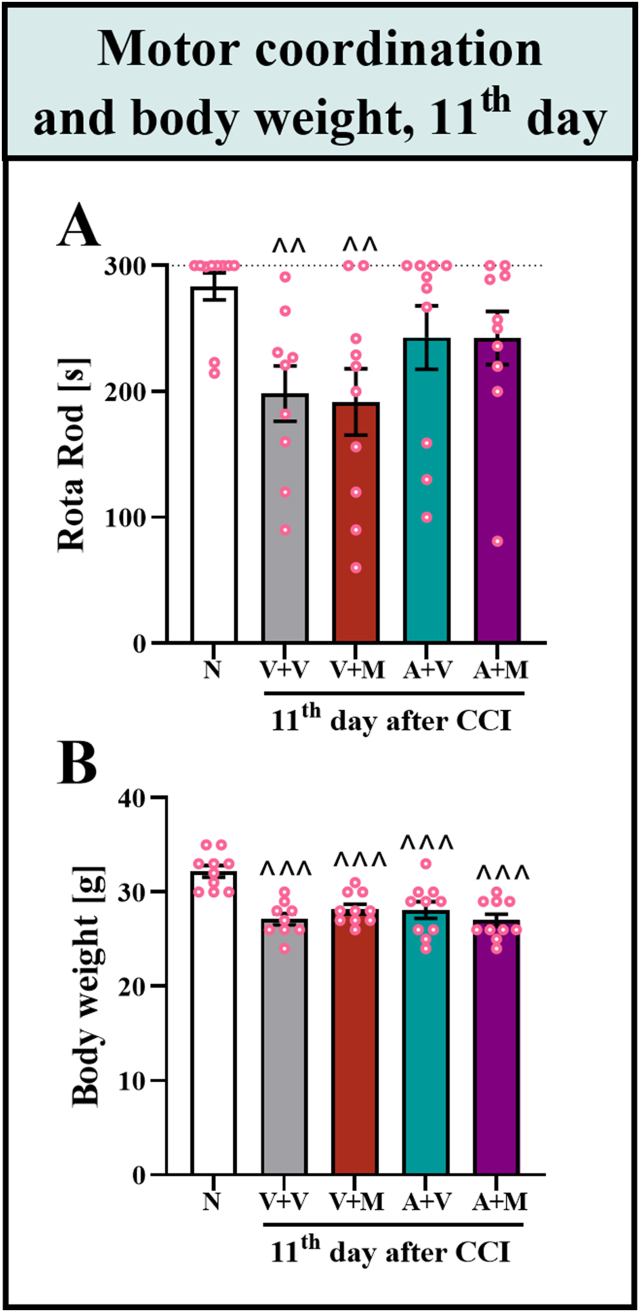
**Effects of the repeated*****i.p.*****administration of the vehicle, astaxanthin (25 mg/kg) or morphine (30 mg/kg) alone or thecombination of astaxanthin with morphine on the motor coordination and body weight of male mice with CCI.** This evaluation was performed on Day 11 as part of the repeated administration study. The rotarod test was conducted 1 h after morphine administration (**A**), and body weight (**B**) measurements were also performed. The results were analyzed using one-way ANOVA with Bonferroni's multiple comparisons *post hoc* test. ^^p<0.01 and ^^^p<0.001 indicate differences between naive and CCI-exposed mice. The dotted line indicate the cutoff values for the rotarod test. Abbreviations: A, astaxanthin; M, morphine; N, naive; V, vehicle.

The effects of astaxanthin and morphine on motor coordination in CCI mice were evaluated on Day 11 as a part of the repeated administration study. At this time point, the analgesic effects of astaxanthin were observed; however, the effectiveness of morphine had already decreased (Figs. 4B and 5B). Importantly, the results revealed that mice subjected to CCI and receiving vehicle or morphine performed worse on the rotating rod than did the naive group (F_4,44_ = 2.96; p = 0.0298); however, in the groups that received astaxanthin, no such effect was observed (Fig. 6A). Additionally, body weight measurements taken on the same day revealed a difference between the naive and CCI-exposed groups, as mice subjected to nerve injury showed a slight reduction in body weight (F_4,44_ = 10.32; p < 0.0001) (Fig. 6B).

### Changes in the mRNA and protein levels of Iba-1, Gfap and cFos in the spinal cord of male mice after repeated, twice-daily morphine or astaxanthin treatment on Day 9 after CCI

**Fig. 7:**
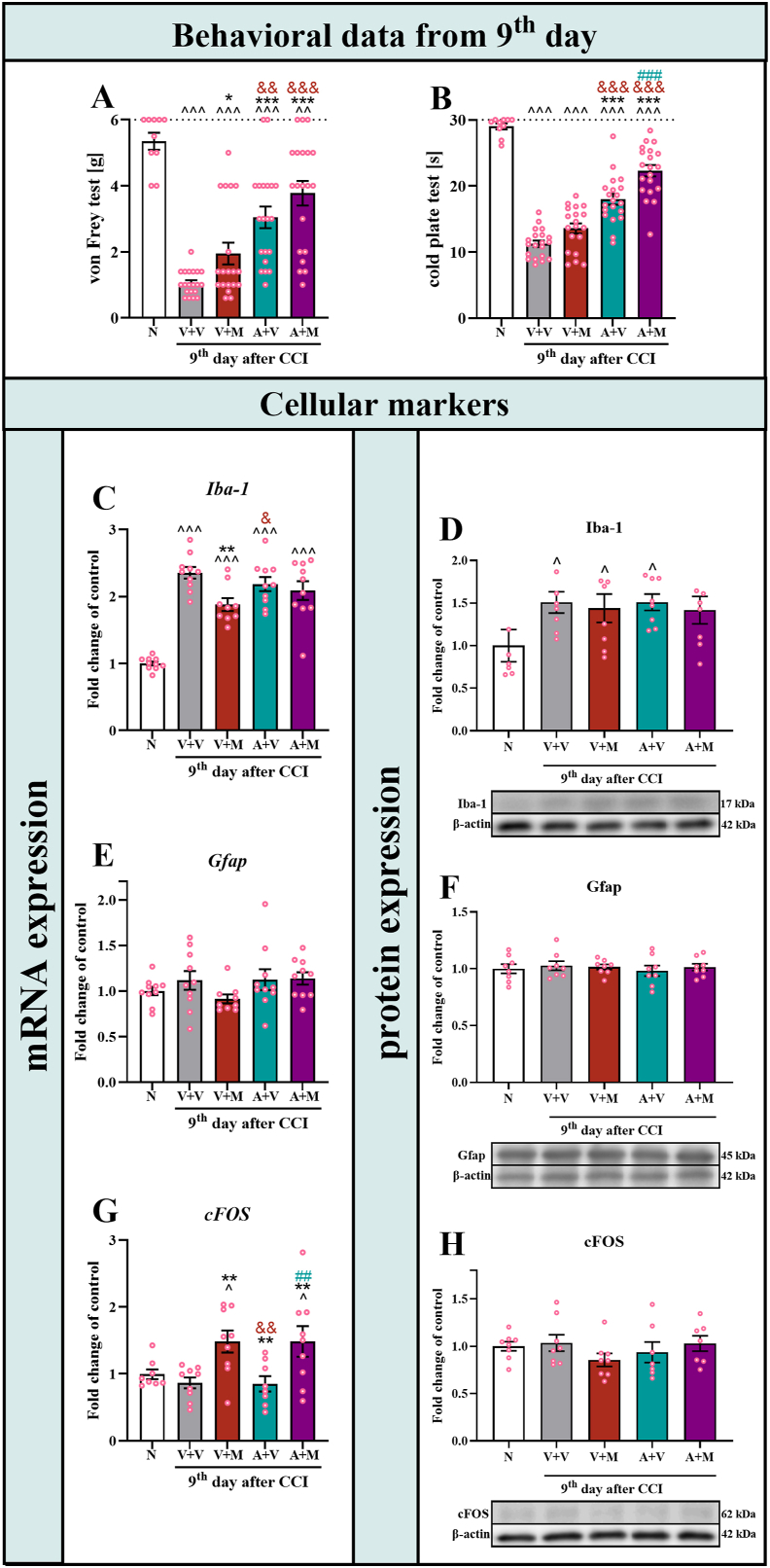
**Analysis of the response of individual animals on Day 9 post-CCI after two daily intraperitoneal injections of the vehicle, morphine (30 mg/kg*****i.p.*****), astaxanthin (25 mg/kg*****i.p.*****) or the combination of astaxanthin with morphine, as measured by von Frey (A) and cold plate (B) tests.** Moreover, the pharmacological effects on the mRNA levels of *Iba-1* (**C**), *Gfap* (**E**) and *cFos* (**G**) were measured *via* RT‒qPCR, and on the protein levels of Iba-1 (**D**), Gfap (**F**) and cFos (**H**) were assessed *via* Western blotting in the spinal cords of CCI-exposed mice on the day 9th and also in naive. The behavioral data are presented as the means ± SEMs (n = 10–20). The biochemical data are presented as the fold changes relative to the control ± SEMs (n = 6–10). The results were analyzed using one-way ANOVA with Bonferroni's multiple comparisons *post hoc* test. Differences between groups are presented as follows:^p<0.05, ^^p^0.01 and ^^^p<0.001 indicate differences compared with the naive mice; ∗p < 0.05, ∗∗p < 0.01 and ∗∗∗p < 0.001 indicate differences compared with the V + V-treated CCI-exposed mice; &p < 0.05, &&p < 0.01, and &&& p < 0.001 indicate differences compared with the V + M-treated CCI-exposed mice; ##p < 0.01 and ###p < 0.001 indicate differences compared with the A + V-treated CCI-exposed mice. The dotted lines indicate the cutoff values for the behavioral tests. Abbreviations: A, astaxanthin; M, morphine; N, naive; V, vehicle; CCI, chronic constriction injury.

On the 9th day after CCI, mechanical and thermal hypersensitivity developed (Fig. 7A and B), as observed in the vehicle-treated group compared with the naive group. Moreover, the analgesic effect of twice-daily repeated morphine administration was diminished in the von Frey test and was no longer detectable in the cold plate test (Fig. 7 A, B). However, the repeated twice daily administration of astaxanthin had good analgesic effects on the results of both the von Frey (F_4,84_ ​= ​24.13; p ​< ​0.0001) (Fig. 7A) and cold plate (F_4,84_ ​= ​71.52; p ​< ​0.0001) (Fig. 7B) tests. Similarly, twice daily injections of astaxanthin with morphine diminished mechanical and thermal hypersensitivity, as observed in both behavioral tests.

On this day of the experiment, changes in the expression of markers of cells involved in nociception were also examined. The results indicate that CCI evokes a significant increase in the expression of the microglia/macrophage marker *Iba-1* mRNA in the spinal cord of the mice, regardless of the treatment (F_4,44_ ​= ​30.02; p ​< ​0.0001) (Fig. 7C)**.** Similarly, at the protein level, nerve injury induced an increase in Iba-1 expression in the spinal cord in all the groups of animals studied, except for the mice that received both morphine and astaxanthin (F_4,34_ ​= ​1.89; p ​= ​0.1335) (Fig. 7D)**.** No changes in the expression of the astrocyte marker Gfap were observed at either the mRNA (F_4,44_ ​= ​1.37; p ​= ​0.2605) (Fig. 7E) or protein (F_4,35_ ​= ​0.22; p ​= ​0.9228) (Fig. 7F) level in any of the studied groups. The expression level of neuronal marker, *cFos*, mRNA in the spinal cord was elevated only in mice treated with morphine or the combination of morphine and astaxanthin, whereas it was lower in mice treated with astaxanthin alone, compared to these two groups (F_4,38_ ​= ​4.79; p ​= ​0.0031) (Fig. 7G), but similar changes were not observed at the protein level of cFos (Fig. 7H).

### Changes in the protein levels of pp38, pERK1/2, pJNK and pNFκB in the spinal cord of male mice after repeated, twice-daily morphine or astaxanthin treatment on Day 9 after CCI

**Fig. 8:**
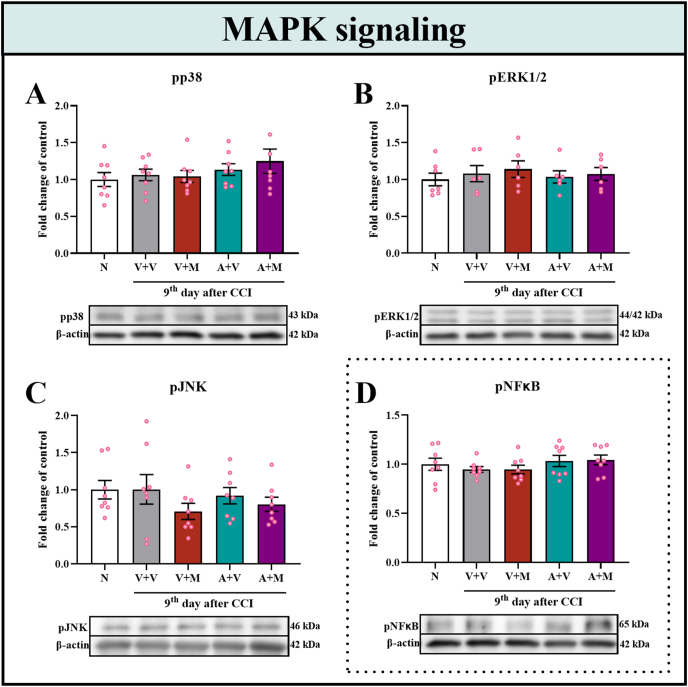
**The pharmacological influence of repeated daily intraperitoneal injections of the vehicle, morphine (30 mg/kg*****i.p.*****), and astaxanthin (25 mg/kg*****i.p.*****) alone and the combination of astaxanthin with morphine on the protein levels of pp38 (A), pERK1/2 (B), pJNK (C) and pNFκB (D) in the spinal cords of CCI-exposed mice on Day 9 and also in naive, as measured by Western blotting.** The data are presented as the fold changes relative to the control ± SEMs (n = 6–8). The results were analyzed using one-way ANOVA with Bonferroni's multiple comparisons *post hoc* test. Abbreviations: A, astaxanthin; M, morphine; N, naive; V, vehicle; CCI, chronic constriction injury.

Western blot analysis of MAPK signaling proteins and the transcription factor NFκB was performed to verify whether these proteins are involved in the analgesic effects of astaxanthin. These factors are of particular interest in neuropathic pain, as both MAPK signaling pathways and NFκB are key regulators of neuroinflammation, glial cell activation, and neuronal sensitization, processes that critically contribute to the initiation and maintenance of neuropathic pain states. However, on Day 9 after CCI, no changes in the spinal cord levels of the pp38 (F_4,35_ ​= ​0.86; p ​= ​0.4950) (Fig. 8A), pERK1/2 (F_4,26_ ​= ​0.30; p ​= ​0.8702) (Fig. 8B), pJNK (F_4,35_ = 0.94; p = 0.4509) (Fig. 8C) and pNFκB (F_4,35_ ​= 0.88; p = 0.4880) (Fig. 8D) proteins were observed regardless of the treatment.

### Changes in the mRNA and protein levels of Sod1, Nrf2 and NR2B in the spinal cord of male mice after repeated, twice-daily morphine or astaxanthin treatment on Day 9 after CCI

**Fig. 9:**
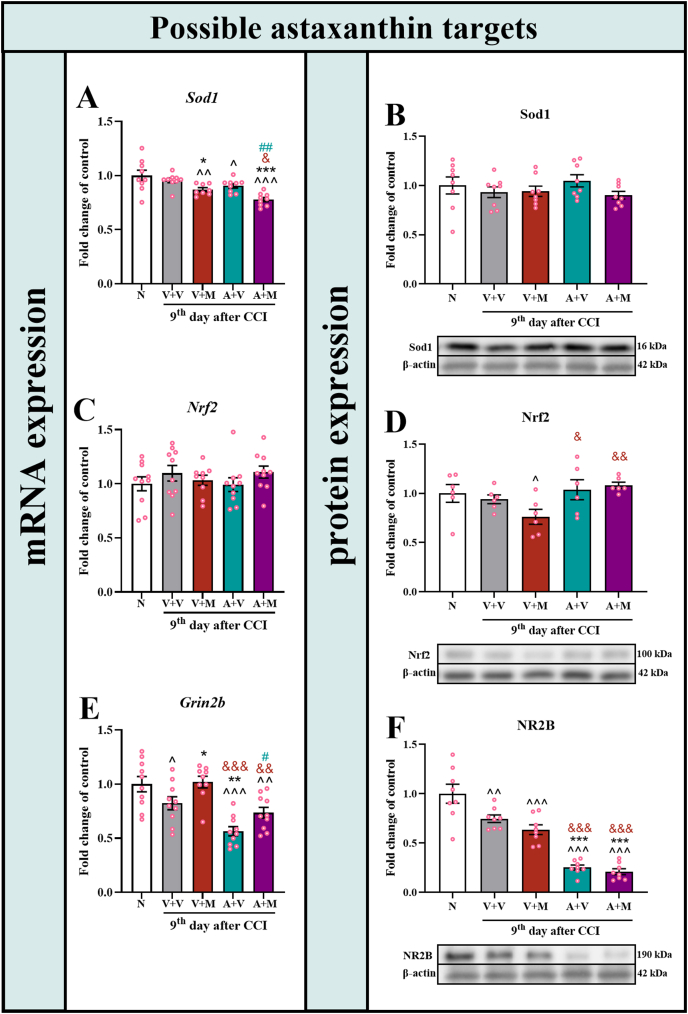
**The pharmacological effects of repeated twice daily intraperitoneal injections of the vehicle, morphine (30 mg/kg*****i.p.*****), astaxanthin (25 mg/kg*****i.p.*****), and the combination of astaxanthin with morphine on the mRNA levels of*****Sod1*****(A),*****Nrf2*****(C) and*****Grin2b*****(E) were measured*****via*****RT‒qPCR, and on the protein levels of Sod1 (B), Nrf2 (D) and NR2B (F) were assessed*****via*****Western blotting on Day 9 in the spinal cords of CCI-exposed mice and also in naive.** The data are presented as the fold changes relative to the control ± SEMs (n = 6–10). The results were analyzed using one-way ANOVA with Bonferroni's multiple comparisons *post hoc* test. Differences between groups are presented as follows: ^p <0.05, ^^p<0.01 and ^^^p<0.001 indicate differences compared with the naive mice; ∗p < 0.05, ∗∗p < 0.01 and ∗∗∗p < 0.001 indicate differences compared with V + V-treated CCI-exposed mice; &p < 0.05, &&p < 0.01 and &&& p < 0.001 indicate differences compared with V + M-treated CCI-exposed mice; #p < 0.05 and ##p < 0.01 indicate differences compared with A + V-treated CCI-exposed mice. Abbreviations: A, astaxanthin; M, morphine; N, naive; V, vehicle; CCI, chronic constriction injury.

To determine whether the mechanisms of action previously proposed for astaxanthin underlie its analgesic effects in neuropathic pain, changes in mRNA and protein expression levels were analyzed. Specifically, the expression of Sod1, the transcription factor Nrf2, and the NR2B subunit of the NMDA receptor was examined, as these molecules are closely related to both the proposed mechanisms of astaxanthin action and key pathways involved in neuropathic pain. Sod1 is a component of the endogenous antioxidant defense system, and its dysfunction is associated with neuronal damage and, as a consequence, the development of hypersensitivity. On Day 9 after CCI, changes in *Sod1* mRNA expression were observed in the mice after treatment with morphine alone, astaxanthin alone or in combination with astaxanthin comparing to naive animals (F_4,39_ ​= ​8.96; p ​< ​0.0001) (Fig. 9A). Furthermore, *Sod1* expression was reduced in animals treated with morphine alone or in combination with astaxanthin, compared to the vehicle-treated group. No changes in Sod1 protein (Fig. 9B) expression were observed in any of the animals, regardless of the treatment (F_4,35_ ​= ​0.94; p ​= ​0.4522).

**Fig. 10:**
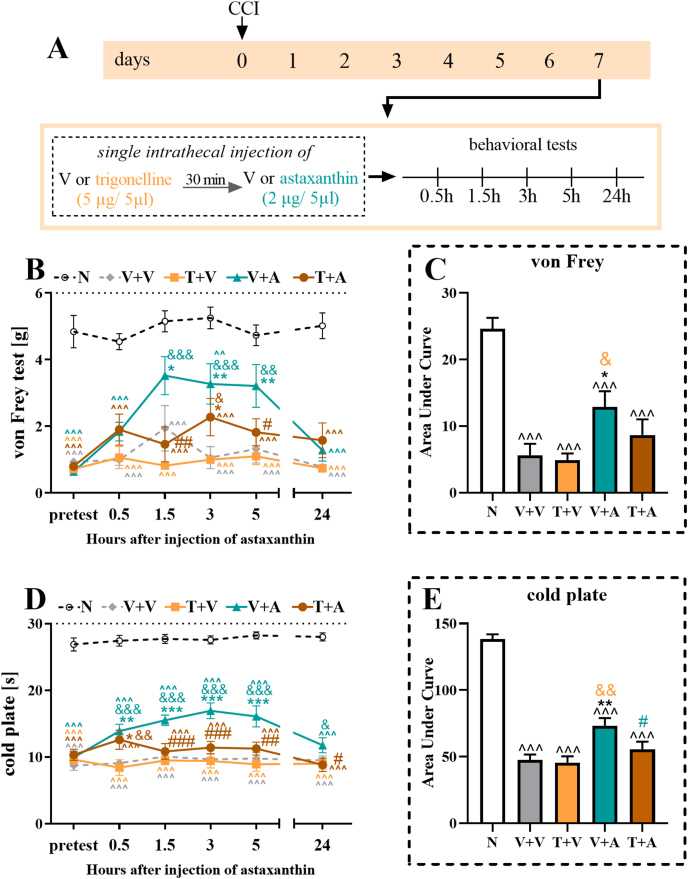
**The influence of a single intrathecal injection of vehicle and astaxanthin (2 μg/5 μl) preceded by the administration of vehicle or trigonelline (5 μg/5 μl) to male mice on Day 7 after CCI, as assessed at 0.5, 1.5, 3, 5 and 24 h after treatment (A), on mechanical and thermal hypersensitivity measured using von Frey (B) and cold plate (D) tests, respectively**. Additionally, the results obtained from the behavioral tests were analyzed as areas under the curves **(C, E)** to visualize overall changes in treatment efficacy. The results were analyzed using one-way ANOVA with Bonferroni's multiple comparisons *post hoc* test. ^^p<0.01, and ^^^p<0.001 indicate differences compared with naive mice; ∗p < 0.05, ∗∗p < 0.01 and ∗∗∗p < 0.001 indicate differences compared with V + V-treated CCI-exposed mice; &p < 0.05, &&p < 0.01, and &&&p < 0.001 indicate differences compared with the T + V-treated CCI-exposed mice; #p < 0.05, ##p < 0.01, and ###p < 0.001 indicate differences compared with the V + A-treated CCI-exposed mice. The dotted lines indicate the cutoff values for the behavioral tests. Abbreviations: A, astaxanthin; N, naive; V, vehicle; CCI, chronic constriction injury; T, trigonelline.

**Fig. 11:**
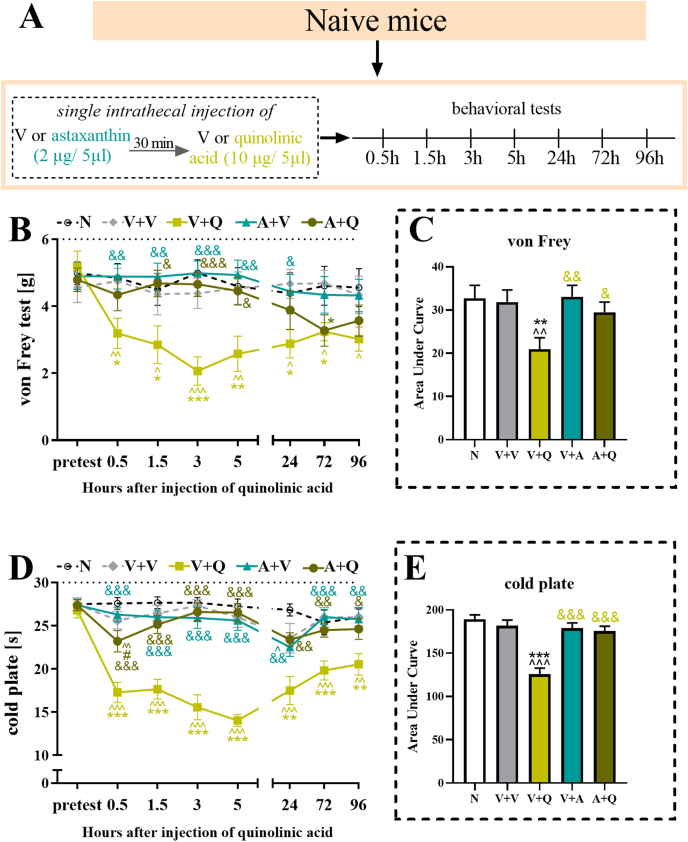
**The influence of a single intrathecal injection of vehicle and quinolinic acid (10 μg/5 μl) preceded by the administration of vehicle or astaxanthin (2 μg/5 μl) to naive male mice on mechanical and thermal hypersensitivity assessed at 0.5, 1.5, 3, 5, 24, 72 and 96 h after treatment (A) using von Frey (B) and cold plate (D) tests, respectively.** Additionally, the results obtained from the behavioral tests were analyzed as areas under the curves **(C, E)** to visualize the overall changes in treatment effectiveness. The results were analyzed using one-way ANOVA with Bonferroni's multiple comparisons *post hoc* test. ^p<0.05, ^^p<0.01, and ^^^ p<0.001 indicate differences compared with naive mice; ∗p < 0.05, ∗∗p < 0.01 and ∗∗∗p < 0.001 indicate differences compared with V + V-treated mice; &p < 0.05, &&p < 0.01, and &&&p < 0.001 indicate differences compared with the V + Q-treated mice; and #p < 0.05 indicates differences compared with the A + V-treated mice. The dotted lines indicate the cutoff values for the behavioral tests. Abbreviations: A, astaxanthin; N, naive; V, vehicle; Q, quinolinic acid.

Nrf2 is a key regulator of the cellular antioxidant response, and its activation mitigates oxidative stress and neuroinflammation, both of which contribute to neuronal hyperexcitability and the development of neuropathic pain. In the case of *Nrf2*, no changes in its mRNA level were observed (F_4,44_ ​= ​0.79; p ​= ​0.5391) (Fig. 9C). Importantly, in terms of protein expression, morphine administered alone led to a decrease in Nrf2 levels comparing to naive animals (F_4,25_ ​= ​2.84; p ​= ​0.0454) (Fig. 9D); in contrast, the administration of astaxanthin alone and in combination with morphine caused higher Nrf2 levels compared with those in the group of mice receiving morphine alone.

Excitatory glutamatergic signaling promotes central sensitization by enhancing neuronal responsiveness to nociceptive input, mainly through activation of the spinal NR2B subunit of NMDA receptors. The expression of its mRNA, *Grin2b*, decreased after CCI in the groups of animals receiving vehicle, astaxanthin alone or in combination with morphine compared with that in naive mice (F_4,44_ ​= ​11.37; p ​< ​0.0001) (Fig. 9E). Moreover, *Grin2b* mRNA expression decreased even more in the group of mice receiving astaxanthin compared to the group receiving the vehicle. In the case of the combined administration of astaxanthin and morphine, the mRNA expression of the *Grin2b* subunit was higher than that in the mice receiving astaxanthin alone but lower than that in the mice receiving morphine alone. Western blot analysis revealed that NR2B protein expression decreased in all animals after CCI, but the greatest decrease was observed in the mice that received astaxanthin alone and those that received astaxanthin in combination with morphine (F_4,35_ ​= ​38.67; p ​< ​0.0001) (Fig. 9F).

### Effects of a single intrathecal injection of astaxanthin preceded by an injection of the Nrf2 inhibitor trigonelline in CCI mice

The Nrf2 inhibitor trigonelline (5 μg/5 ​μl) was administered 30 ​min before astaxanthin (2 μg/5 ​μl) on Day 7 after CCI to evaluate whether activation of the Nrf2 signaling pathway is involved in the antinociceptive effects of astaxanthin (Fig. 10A). The von Frey test results indicated that trigonelline administration did not affect mechanical hypersensitivity in mice with CCI at any time point examined (Fig. 10B). Astaxanthin administration led to a reduction in hypersensitivity at all examined time points, except at 0.5 and 24 ​h post-administration. Importantly, the administration of trigonelline before astaxanthin partially blocked the effectiveness of astaxanthin in reducing hypersensitivity to mechanical stimuli, as shown by the results of the von Frey test at 1.5 ​h (F_4,44_ ​= ​13.47; p ​< ​0.0001) and 5 ​h (F_4,44_ ​= ​13.45; p ​< ​0.0001) after injections. Two-way repeated-measures ANOVA confirmed a significant interaction between the investigated treatment and the tested time points (F_20,268_ ​= ​2.01; p ​= ​0.0071, Fig. 10B).

Trigonelline administration also did not affect hypersensitivity to thermal stimuli, as indicated by the cold plate test results (Fig. 10D), unlike astaxanthin, which evoked analgesic effects at all of the time points examined, except 24 ​h. The administration of trigonelline before astaxanthin blocked the effectiveness of astaxanthin in reducing hypersensitivity to thermal stimuli, as shown at 1.5 ​h (F_4,44_ ​= ​74.15; p ​< ​0.0001), 3 ​h (F_4,45_ ​= ​67.84; p ​< ​0.0001), 5 ​h (F_4,44_ ​= ​58.06; p ​< ​0.0001) and 24 ​h (F_4,45_ ​= ​90.10; p ​< ​0.0001) after administration. Two-way repeated-measures ANOVA confirmed a significant interaction between the investigated treatment and the tested time points (F_20,270_ ​= ​1.81; p ​= ​0.0193, Fig. 10D).

Analysis of AUC values for the von Frey test indicated that trigonelline did not affect the efficacy of astaxanthin (Fig. 10C), however AUC analysis for the cold plate test showed that trigonelline attenuated the analgesic effects of astaxanthin (F_4,15_ ​= ​64.74; p ​< ​0.0001) (Fig. 10E).

### Effects of a single intrathecal injection of quinolinic acid preceded by an astaxanthin injection in naive mice

The NMDA receptor agonist quinolinic acid (10 μg/5 ​μl) was administered to naive mice 30 ​min after astaxanthin (2 μg/5 ​μl) to evaluate whether NMDA signaling is involved in the antinociceptive effects of astaxanthin (Fig. 11A). As indicated by the von Frey test results, the administration of quinolinic acid alone induced long-lasting hypersensitivity to mechanical stimuli, as observed at all the examined time points, with the most pronounced effect observed 3h (F_4,44_ ​= ​9.36; p ​< ​0.0001) after injection, whereas the injection of astaxanthin alone did not affect the sensitivity of naive mice (Fig. 11B). Importantly, the administration of astaxanthin prior to the quinolinic acid injection partially blocked the occurrence of hypersensitivity to mechanical stimuli, as shown by the test results between 1.5 ​h (F_4,44_ ​= ​2.60; p ​= ​0.0488), and 5 ​h (F_4,44_ ​= ​3.66; p ​= ​0.0116) after treatment. Two-way repeated-measures ANOVA did not confirmed a significant interaction between the investigated treatment and the tested time points (F_28,353_ ​= ​1.01; p ​= ​0.4517, Fig. 11B).

Quinolinic acid administration also induced hypersensitivity to thermal stimuli, which was still observed at 96 ​h after treatment (F_4,43_ ​= ​4.00; p ​= ​0.0076). Astaxanthin administration did not affect the response in the healthy animals, whereas its administration (Fig. 11D) prior to quinolinic acid blocked the occurrence of hypersensitivity to the thermal stimulus at 0.5 (F_4,44_ ​= ​16.89; p ​< ​0.0001), 1.5 ​h (F_4,44_ ​= ​18.22; p ​< ​0.0001), 3 ​h (F_4,44_ ​= ​23.80; p ​< ​0.0001), 5 ​h (F_4,44_ ​= ​30.97; p ​< ​0.0001), 24 ​h (F_4,44_ ​= ​7.12; p ​= ​0.0002) and 72 ​h (F_4,43_ ​= ​5.60; p ​= ​0.0010) after treatment. Two-way repeated-measures ANOVA confirmed a significant interaction between the investigated treatment and the tested time points (F_28,351_ ​= ​3.11; p ​< ​0.0001, Fig. 11D).

An analysis of the AUCs of both behavioral tests revealed that the groups receiving astaxanthin did not exhibit pain-related behaviors after treatment (von Frey - F_4,20_ ​= ​3.49; p ​= ​0.0256; cold plate - F_4,20_ ​= ​17.01; p ​< ​0.0001) (Fig. 11C–E).

## Discussion

The findings of this study provide new insights into the potential use of astaxanthin for the treatment of neuropathic pain. These experiments demonstrate that a single intraperitoneal injection of astaxanthin reduces mechanical and thermal hypersensitivity to a similar extent in both male and female mice in the CCI model of neuropathic pain. Importantly, this study provides the first evidence that systemic, repeated administration of astaxanthin in combination with morphine from the onset of treatment effectively delays the development of opioid tolerance and prevents motor impairments in the CCI model of neuropathic pain. Furthermore, even when administered after morphine tolerance has already developed, astaxanthin continues to exert beneficial effects on pain-related behaviors. Notably, twice-daily intraperitoneal administration of astaxanthin, initiated one day prior to nerve injury, results in a sustained and robust analgesic effect. Strikingly, in this treatment regimen, astaxanthin's therapeutic efficacy surpasses that of morphine alone in the neuropathic pain model employed in this study. Our biochemical analyses demonstrate that astaxanthin modulates important molecular targets implicated in neuropathic pain. Specifically, astaxanthin increases Nrf2 protein levels while significantly reducing the expression of the NR2B subunit of the NMDA receptor in the spinal cord. These molecular changes suggest two potential mechanisms underlying its analgesic action, further supported by our pharmacological findings. Notably, the ability of astaxanthin to attenuate CCI-induced mechanical and thermal hypersensitivity was abolished by coadministration of trigonelline, a known Nrf2 inhibitor. Additionally, in naive mice, astaxanthin completely prevented hypersensitivity induced by quinolinic acid, an NMDA receptor agonist. Collectively, these results suggest that the analgesic efficacy of astaxanthin involves both activation of the Nrf2 and inhibition of NMDA receptor signaling *via* NR2B subunit modulation. Thus, astaxanthin may represent a promising therapeutic candidate for the treatment of neuropathic pain in the future.

Neuropathic pain, resulting from nerve injury, is a chronic condition that often exhibits resistance to standard therapeutic approaches [3]. In recent years, there has been growing interest in natural compounds with analgesic potential. Among these, astaxanthin has emerged as a promising candidate [9] due to its diverse biological activities and demonstrated efficacy in alleviating both inflammatory and neuropathic pain [57,58,60,[79], [80], [81]]. In our previous study, we demonstrated that a single intrathecal administration of astaxanthin effectively reduced hypersensitivity in male mice subjected to CCI [57]. The present findings extend these observations by showing, for the first time, that a single intraperitoneal injection of astaxanthin significantly attenuates CCI-induced hypersensitivity, with comparable efficacy observed in both male and female mice. The results obtained are consistent with those reported in 2024 in a model of diabetic neuropathy [60]. Our study presents novel finding: repeated intraperitoneal administration of astaxanthin produced a significant analgesic effect from the beginning to the last day of the experiments. We subsequently investigated whether the concomitant administration of astaxanthin and morphine could enhance their analgesic efficacy in the context of neuropathic pain. Given that repeated morphine use is associated with numerous adverse effects, it is currently considered a third-line treatment option for neuropathic pain [4,82]. The emerging problem in neuropathy is the lower efficacy of opioids and rapid development of morphine tolerance, which requires increasing doses to maintain a satisfactory level of pain relief in patients [83]. Similarly, in our behavioral experiments, we observed a gradual decrease in morphine analgesia after repeated administration in the CCI model; morphine diminished mechanical and thermal hypersensitivity until Days 8–10. Importantly, symptoms of opioid-induced hyperalgesia were also observed at later time points, which is consistent with the available literature [84,85]. To date, the results published in 2023 showed that a single intrathecal injection of astaxanthin in combination with morphine enhanced its analgesic effects on a CCI model [57]. Consequently, we aimed to advance our investigations through repeated intraperitoneal coadministration of the drugs. From a clinical perspective, systemic drug administration is highly important in experimental studies. Notably, the administration of astaxanthin to mice that had already developed tolerance to morphine resulted in partial pain relief and effectively mitigated the onset of opioid tolerance. Intriguingly, in this experimental framework, astaxanthin administered alone yielded significant analgesic effects. In a subsequent experimental design, the coadministration of astaxanthin with morphine during the early phase of neuropathy—employing a preemptive and then repeated injections approach—significantly delayed the development of morphine tolerance. This intervention resulted in a robust and sustained analgesic effect, as demonstrated by a prolonged reduction in both mechanical and thermal hypersensitivity that persisted for the entire 14-day observation period. Notably, repeated administration of astaxanthin maintained its analgesic efficacy over time and exhibited superior therapeutic properties compared to morphine in the neuropathic pain model employed in this study. These findings are further supported by our previous results obtained in diabetic neuropathic pain model, where astaxanthin similarly provided consistent and long-lasting pain relief without signs of diminished effectiveness [60]. Importantly, the results reported by other authors indicate that the oral administration of astaxanthin for three weeks also reduces hypersensitivity in a mouse model of spared nerve injury [56]. Additionally, those authors proved that astaxanthin also attenuated depressive behaviors [56,86], which is important because patients with neuropathic pain often suffer from mood disorders, and antidepressants are used for therapy [87,88]. Notably, astaxanthin has been shown to exert neuroprotective effects following spinal cord and brain injury [51,81,89], which is in agreement with our results showing that astaxanthin prevents the development of motor disturbances after nerve injury. In conclusion, both our findings and those of previous studies indicate that astaxanthin represents a promising therapeutic candidate for the management of neuropathic pain of diverse etiologies, with evidence suggesting that its long-term administration may confer sustained analgesic benefits.

In subsequent research endeavors, we sought to elucidate the underlying mechanisms through which astaxanthin exerts its notable efficacy in alleviating neuropathic pain, as well as its role in enhancing the effectiveness of morphine. Our investigation aimed to validate the hypotheses proposed by previous studies concerning the potential mechanisms of action of astaxanthin [9]. In vitro studies have shown that astaxanthin may inhibit the polarization of LPS-activated primary microglia [90] and the BV-2 microglial cell line [91] from the M1 phenotype to the M2 phenotype [53]. In addition, the oral administration of astaxanthin decreases the number of Iba1-positive cells in the hippocampus after systematic LPS administration [91]. Therefore, astaxanthin may reduce the degree of microglial/macrophage activation in a neuropathic pain model, as increasing evidence indicates that these cells are highly activated in the spinal cord and contribute to the development of neuropathic pain and opioid tolerance [5,92]. Microglia/macrophages are considered crucial for generating and maintaining neuropathic pain, and their interactions with neurons and astroglia are essential for neuroinflammation [5,92]. Our studies confirmed that CCI induces the upregulation of spinal Iba-1 levels, which is consistent with the literature [64,78,85]. Unexpectedly, astaxanthin did not alter the expression levels of protein markers associated with microglia/macrophages (Iba-1), neurons (cFos), or astrocytes (Gfap) on Day 9 post-CCI. This finding suggests that, contrary to previous reports, modulation of these cell types may not represent the principal mechanism underlying the analgesic effects of astaxanthin in the treatment regimen employed in this study. The data published thus far indicate that astaxanthin can also inhibit MAPKs [58,[93], [94], [95]], which are important in the intracellular modulation of the nociceptive response [5,9,92]. Selective inhibitors of p38 (SB203580 and FR167653) [20,22,96,97], ERK1/2 (U0126, PD98059, and PD198306) [21,24,34,98,99], and JNK (D-JNKI-1JNK and SP600125) [12,100] reduce hypersensitivity in several neuropathic pain models. Moreover, astaxanthin relieves pain by inhibiting p38 and ERK1/2 activation in mouse models of spinal nerve ligation [58] and complete Freund's adjuvant injection [79]. However, in our experiments, the repeated intraperitoneal administration of astaxanthin did not affect the phosphorylation of p38, pERK1/2, pJNK or pNFκB; at least, we did not observe such an effect on Day 9. Data from the literature indicate that p38 can be correlated with microglial activation [20], but in our experiments, astaxanthin did not affect either pp38 or Iba-1 levels; therefore, we assume that the analgesic properties of astaxanthin observed on Day 9 are related to other mechanisms.

Given that the analgesic effects of astaxanthin observed in the CCI model do not appear to result from its influence on the previously mentioned factors, we sought to examine its effect on Nrf2, as astaxanthin has been reported to activate this transcription factor [101]. Under physiological conditions, Nrf2 is located in the cytoplasm through its interaction with Kelch-like ECH-associated protein 1 (Keap1) [[102], [103], [104]]. In response to oxidative stress, Nrf2 dissociates from Keap1, translocates to the nucleus, and binds to the antioxidant response element known as ARE, a regulatory enhancer sequence, thereby modulating the expression of approximately 250 genes involved in the upregulation of various antioxidant and cytoprotective enzymes [105]. Moreover, activation of Nrf2 has been shown to mitigate mitochondrial dysfunction [74,[106], [107], [108]]. Importantly, Nrf2 downstream interactions may lead to the transcription of several antioxidant enzymes that relieve pain in animal neuropathic pain models [74,[107], [108], [109], [110]]. This finding is consistent with our previous pharmacological results, in which bardoxolone methyl, an Nrf2 activator [111,112], significantly reduced mechanical and thermal hypersensitivity in mice subjected to CCI [57]. Similar effects have also been observed by other researchers on diabetic [113] and ischemic optic [114,115] neuropathy models. Importantly, astaxanthin was recently shown to provide neuroprotection *via* the Nrf2/HO-1 pathway after brain injury [89]. We observed analogous beneficial effects, as astaxanthin effectively mitigated the motor disturbances induced by CCI. Notably, studies from 2011 demonstrated that spinal cord motor neurons exhibit increased susceptibility to oxidative stress, as evidenced by experiments using a Sod1 inhibitor, which exerts growth-inhibitory effects. Importantly, this heightened vulnerability was effectively attenuated by astaxanthin treatment [116]. However, under the administration regimen we employed, no changes in Sod1 protein expression levels were observed following astaxanthin treatment. We therefore aimed to verify whether the observed beneficial effects of astaxanthin are directly linked to its modulation of Nrf2. Findings from our pharmacological studies provide evidence that Nrf2 may indeed play a pivotal role in mediating the analgesic properties of astaxanthin. Behavioral assessments revealed that intrathecal administration of astaxanthin significantly alleviates both mechanical and thermal hypersensitivity induced by CCI, an effect that was notably abolished by co-administration of the Nrf2 inhibitor trigonelline. These results are particularly important, as they demonstrate for the first time the direct involvement of Nrf2 in the antinociceptive action of astaxanthin in a neuropathic pain model. Furthermore, our findings suggest that Nrf2 may also contribute to the enhanced analgesic efficacy of morphine observed following astaxanthin treatment. This possibility is supported by the latest published data indicating that Nrf2 activators, bardoxolone methyl [57], 5-fluoro-2-oxindole [117] and sulforaphane [118], enhance morphine analgesia. Moreover, our biochemical data revealed decreased Nrf2 protein level after repeated morphine administration, suggesting that this protein is important in the development of tolerance to the analgesic effects of morphine. Our current research provides the first evidence that the repeated administration of astaxanthin with morphine is able to delay tolerance development, which may be related to the increased level of the Nrf2 protein observed on Day 9 after CCI. Importantly, astaxanthin evokes stronger analgesic effects than this opioid drug alone does; in our opinion, this result may be due, among other reasons, to its beneficial effect on Nrf2. Considering the available literature and the findings of subsequent studies, we believe that, from a clinical point of view, the regulation of Nrf2 in neuropathy is extremely important, and its pharmacological modulation by astaxanthin may contribute to more effective therapies.

Interestingly, an additional and distinct mechanism underlying the action of astaxanthin has recently been proposed. *In silico* molecular docking studies have demonstrated that astaxanthin fits into the inhibitory binding pocket of the NR2B subunit of the NMDA receptor, a key component involved in nociceptive signaling [80]. NR2B plays a crucial role in neuropathic pain mechanisms, exhibiting the highest expression among NR2 subunits in the spinal dorsal horn [119]. Importantly, our study revealed a strong downregulation of NR2B protein levels in the spinal cord following astaxanthin administration, consistent with previous observations in rats after spinal cord injury [81]. Moreover, similar effects have been reported with selective NR2B antagonists, which also exhibit analgesic properties in animal pain models [120]. Behavioral investigation of NR2B function is limited by the absence of a selective agonist; therefore, we employed quinolinic acid, an endogenous NMDA receptor ligand acting on both NR2B and NR2A subunits [121]. Previous studies, however, have shown that NR2A-deficient mice develop neuropathic pain comparable to wild-type mice [122], whereas overexpression of NR2B enhances pain-related behaviors [42]. Moreover, NR2A appears to be absent from the peripheral terminals of primary afferent neurons [119]. Notably, quinolinic acid is synthesized within the spinal cord during the development of neuropathic pain, primarily by activated microglia and macrophages [121], and the strong activation/infiltration/proliferation of these cells after CCI was confirmed by our Western blot analysis. Under physiological conditions, quinolinic acid concentration in the nervous system is typically below 100 ​nM; however, in pathological states, levels can rise significantly, reaching 500–1200 ​nM [123]. Moreover, a recent study involving over 17 ​000 patients with chronic pain identified quinolinic acid as one of the most frequently elevated marker associated with pain states [124]. Our study provides the first evidence that intrathecal administration of astaxanthin, followed by quinolinic acid, prevents the development of hypersensitivity to mechanical and thermal stimuli, indicating the involvement of the NMDA receptor in the analgesic mechanism of astaxanthin. Taking into account *in silico* data and the consistency of our results with those obtained using selective NR2B antagonists [120,125,126], this subunit appears to play a key role in astaxanthin's analgesic effects in neuropathy. Current research is focused on the use of NMDA receptor antagonists as key therapeutic agents for pain relief [127]. The analgesic properties of antagonists, such as ketamine, memantine, dextromethorphan, and magnesium, are the subject of intensive experimental and clinical research [128]. Ketamine is frequently used to treat various chronic pain syndromes, especially those that have a neuropathic component [129]. In addition, our biochemical studies revealed that astaxanthin, like ketamine, reduces the expression of the NR2B subunit in the spinal cord, which is related to reduced pain hypersensitivity [130]. Moreover, since the blockade of the NMDA receptor subunit NR2B by Ro 256 ​981 has already been shown to reduce the development of morphine tolerance [40], we hypothesize that by blocking NR2B, astaxanthin improve the efficacy and prolong the duration of action of morphine in our experiments. Systemic administration of astaxanthin in a mouse model of diabetic neuropathy has been shown to prevent both the development of hypersensitivity and the onset of morphine tolerance [60]. In light of these previously published findings, along with the results of the present study, we propose that astaxanthin - through its ability to inhibit the NR2B subunit of the NMDA receptor - may represent a promising therapeutic strategy for the treatment of neuropathic pain. Nonetheless, further preclinical and clinical investigations are warranted to fully elucidate its efficacy and mechanism of action.

In conclusion, the management of neuropathic pain continues to pose a significant challenge for both patients and healthcare professionals. Individuals affected by this condition often experience chronic symptoms that markedly impair quality of life, while available pharmacological therapies frequently offer only limited and transient relief. In this context, the growing interest in natural compounds as therapeutic agents has brought astaxanthin to the forefront as a promising candidate. Importantly, from a translational perspective, our findings demonstrate that astaxanthin administration not only provides a sustained reduction in pain hypersensitivity but does so without inducing motor deficits. Moreover, it effectively prevents the development of morphine tolerance in a neuropathic pain model. These behavioral effects, together with our biochemical analyses in a murine model, support the conclusion that both activation of the Nrf2 signaling pathway and inhibition of the NR2B subunit of the NMDA receptor are key mechanisms underlying the analgesic properties of astaxanthin. This dual action highlights a complex, multitarget mechanism of analgesia, underscoring astaxanthin's potential as a unique therapeutic tool for addressing neuropathic pain and possibly other pain-related conditions. Furthermore, astaxanthin is already approved as a dietary supplement with a well-established safety profile, further supporting its clinical viability [131]. Of particular significance, two recent clinical studies have reported that astaxanthin-based preparations, PeaNoc XL [132] and FlexPro MD® [133], effectively contribute to the reduction of pain and joint stiffness, highlighting astaxanthin potential therapeutic value. Taken together, our results indicate that astaxanthin holds considerable promise as a therapeutic agent for the treatment of neuropathic pain. Nonetheless, additional preclinical studies and rigorously designed clinical trials are essential to confirm its efficacy and safety across diverse neuropathic conditions.

## CRediT authorship contribution statement

**Katarzyna Ciapała:** Conceptualization, Data curation, Formal analysis, Investigation, Methodology, Validation, Visualization, Writing – original draft, Writing – review & editing. **Katarzyna Pawlik:** Conceptualization, Data curation, Formal analysis, Investigation, Methodology, Validation, Writing – review & editing. **Agata Ciechanowska:** Formal analysis, Investigation, Methodology, Writing – review & editing. **Wioletta Makuch:** Data curation, Formal analysis, Investigation, Methodology, Writing – review & editing. **Joanna Mika:** Conceptualization, Data curation, Formal analysis, Funding acquisition, Investigation, Project administration, Supervision, Writing – original draft, Writing – review & editing.

## Data availability statement

The data presented in this study are available upon request from the corresponding author.

## Ethical approval

All applicable international, national, and institutional guidelines for the care and use of animals were followed. The number of animals was limited to the necessary minimum. The experiments were carried out according to the recommendations and standards of the International Association for the Study of Pain and the National Institutes of Health Guide for the Care and Use of Laboratory Animals and were approved by the II Ethical Committee of the Maj Institute of Pharmacology of the Polish Academy of Sciences (permission numbers: LKE 75/2023; 18/2023; 153/2023; 242/2024; and 243/2024).

## Funding

This research was funded by grants from the 10.13039/501100004442National Science Centre, Poland, 10.13039/100025296OPUS 22 2021/43/B/NZ7/00230 and statutory funds from the Maj Institute of Pharmacology 10.13039/501100004382Polish Academy of Sciences.

## Declaration of competing interest

The authors declare that they have no known competing financial interests or personal relationships that could have appeared to influence the work reported in this paper.
